# Technological improvements or climate change? Bayesian modeling of time-varying conformance to Benford’s Law

**DOI:** 10.1371/journal.pone.0213300

**Published:** 2019-04-12

**Authors:** Junho Lee, Miguel de Carvalho

**Affiliations:** School of Mathematics, University of Edinburgh, Edinburgh, United Kingdom; Auburn University - Harrison School of Pharmacy, UNITED STATES

## Abstract

We develop a Bayesian time-varying model that tracks periods at which conformance to Benford’s Law is lower. Our methods are motivated by recent attempts to assess how the quality and homogeneity of large datasets may change over time by using the First-Digit Rule. We resort to a smooth multinomial logistic model which captures the dynamics governing the proportion of first digits, and apply the proposed model to global tropical cyclone tracks over the past two centuries. Our findings indicate that cumulative technological improvements may have only had a moderate influence on the homogeneity of the dataset, and hint that recent heterogeneity could be due to other drivers.

## Introduction

Benford’s Law is an empirical observation on the distribution of first digits of numerical data discovered by [1] and [2]. The law states that, in many situations of applied interest, the frequency of the first digit of numbers follows a logarithmically decreasing distribution—even though it is generally believed that the probability of occurrence of each number is equally likely. The probability that the first non-zero digit begins with a number *d* follows a logarithmic distribution given by

$$\begin{array}{c} {\text{p}}_{d}=\text{P}\left(D=d\right)=\log_{10}(1+\frac{1}{d}),\;d=1,\ldots ,9, \end{array}$$ (1)

where *D* is the first significant digit of a random variable. The probability of the significant leading digit equal to 1, for example, is calculated as approximately 0.301, and then the probability of the leading digit equals *d* gets smaller as *d* increase, up to where the probability of the leading digit 9 equals to only 0.046. A wide variety of datasets, especially a collection of datasets, have been reported to conform to Benford’s Law. A statistical foundation of its universality was presented by [3]. Since the peculiar law of first digits uncovered, a battery of studies showed that large classes of quantities in different disciplines from both natural phenomena and social activities are expected to follow the First-Digit Rule, and therefore it can be used for detecting structural changes or irregularities from various applications [4, 5].

This paper devises a Bayesian time-varying model that tracks periods at which conformance to Benford’s Law is lower. Our methods are motivated by recent attempts to assess how the quality and homogeneity of large datasets may change over time by using the First-Digit Rule (e.g. [6–8]). As we show in the numerical studies in the Supplementary Materials (S1 File), the empirical-based approach by [8] suffers often from bias (cf Fig 4, S1 File)—thus questioning some of their key empirical findings. Our Bayesian smooth multinomial logistic model is however accurate (cf numerical studies in S1 File), and it is tailored—by construction—for capturing the dynamics governing the proportion of first digits. We apply the proposed model to global tropical cyclone tracks over the past two centuries, and compare our empirical findings with those of [8]. An application of our model indicates that cumulative technological improvements may have only had a moderate influence on the homogeneity of the dataset. Indeed, although technological improvements are cumulative we find that the most recent heterogeneity levels actually tend to be higher than the ones from 1842 to 1890 (cf Fig 4, below); this finding seems to be in contradiction with [8] (cf Fig 5 in their paper), possibly due to the above-mentioned bias issue. Finally, while we center the article on the tropical cyclone application, our Bayesian time-varying approach has the potential to be employed on other contexts where the target is on learning about the dynamics governing conformance to Benford’s Law—including fraud analysis.

The paper is organized as follows. We first introduce our motivating global tropical cyclone data and provide preliminary statistics on their conformance to Benford’s Law. The next section describes our proposed Bayesian multinomial logistic smoothing model along with details on prior specification and on inference. The homogeneity of cyclone data is then analyzed by inspecting dynamics of the first-digit distribution. Lastly, we discuss data homogeneity and other issues based on the results. For the convenience of exposition, specific details surrounding numerical experiments on the model and relevant code in R [9] are left to the S1 File.

## Materials and methods

### Global tropical cyclones (GTC) dataset

**Fig 1:**
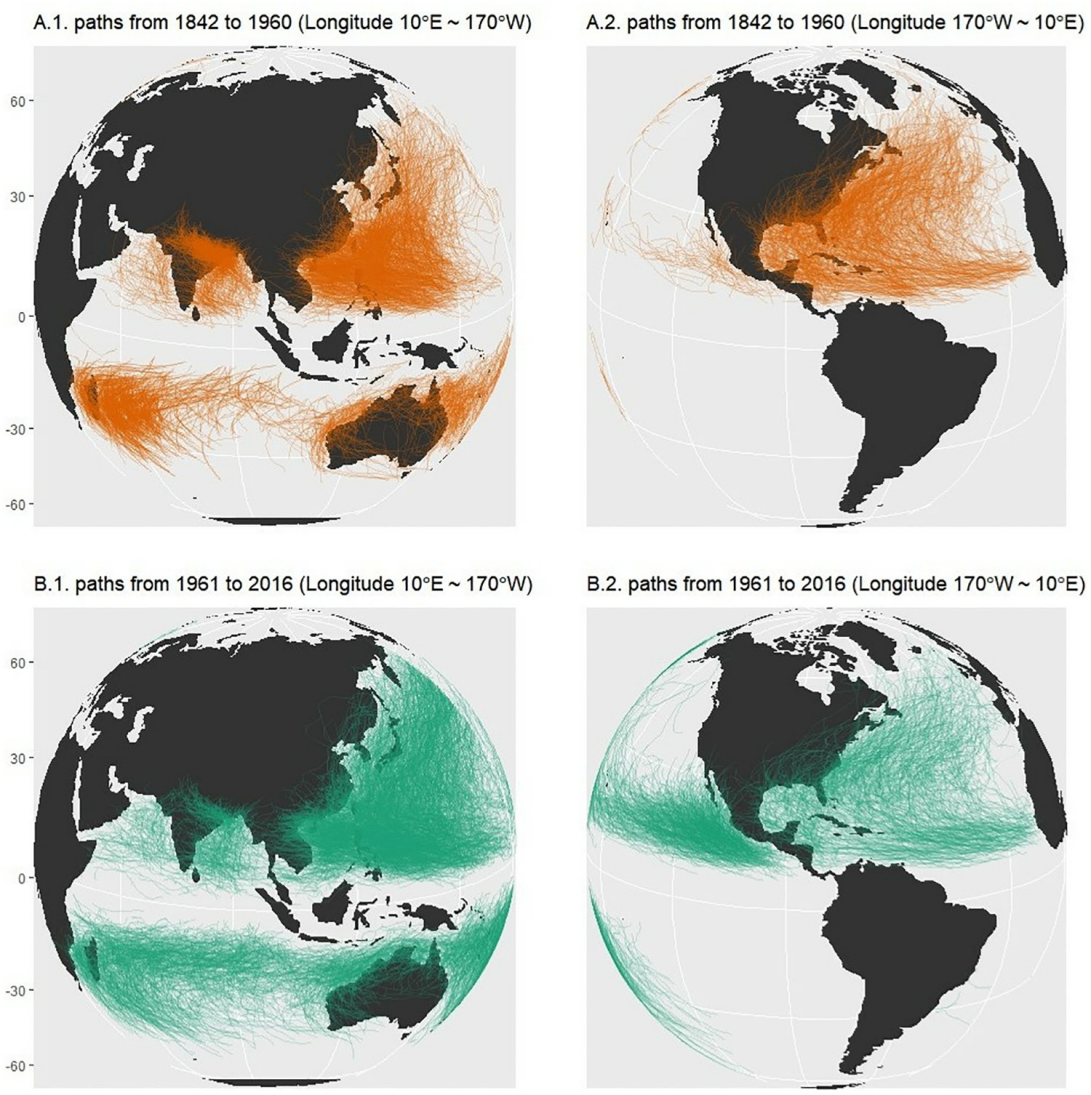
Map of the global tropical cyclones tracks from International Best Track Archive for Climate Stewardship (IBTrACS). (Top) paths from 1842 to 1960; (Bottom) paths from 1961 to 2017;(Left) Map projection on longitude 10°E ∼170°W; (Right) Map projection on Longitude 170°W ∼10°E.

The GTC dataset provides information on the distribution, frequency, and intensity of tropical cyclones worldwide, which is collected as a project of International Best Track Archive for Climate Stewardship (IBTrACS). The dataset includes a register of tropical cyclones since 1842, and is available from the website of IBTrACS (https://www.ncdc.noaa.gov/ibtracs). It has multiple observed records of each cyclone such as geographical location, temperature, and wind speed. As of May of 2018, a total of 348,703 traveled locations are recorded with the corresponding climatic information. Fig 1 presents the traveled path of each cyclone in the dataset over the entire period.

Apart from the intrinsic heterogeneity of tropical cyclones, there has been a debate on the quality of early records in the dataset for assessing the influence of climate change on the occurrence of tropical cyclones [10, 11].

**Fig 2:**
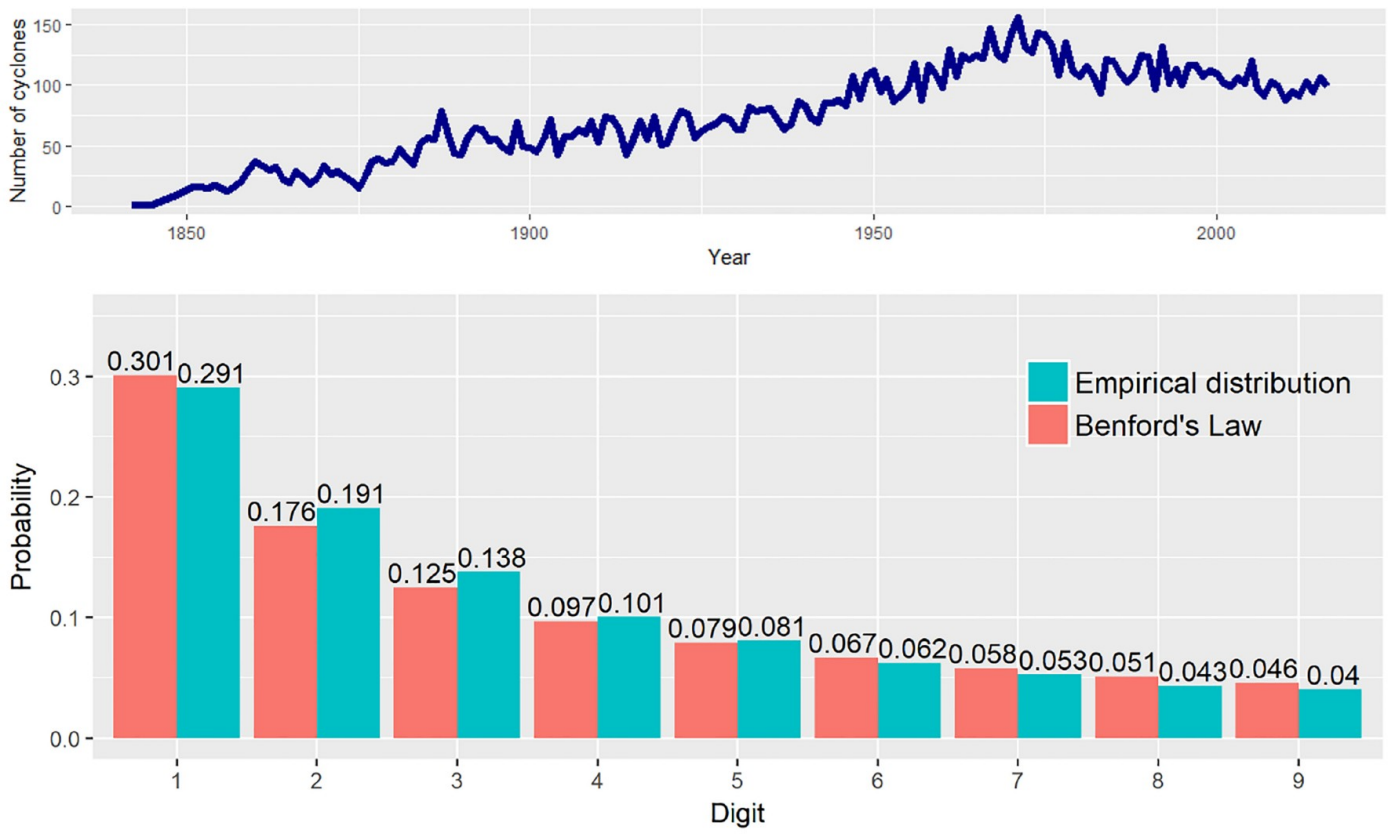
Descriptive statistics for GTC data. Above: Frequency of tropical cyclones from 1842–2016. Below: Empirical first-digit distribution of the traveled distance (in meters) per cyclone is represented (red) along with the corresponding probability mass for Benford’s distribution (blue).

We retrieve observed location records of each cyclone from 1842 to 2016 in the GTC dataset and then trace a geometric path by connecting points which each cyclone traveled. We measure distance per cyclone in meters along the path using the geosphere package [12] from the R programming language. Except for the small cyclones with a single geographical location (latitude/longitude), we obtain 12,741 observations of traveled distances in total; Fig 2 depicts the frequency of tropical cyclones over the period under analysis. This allows us to analyze dynamics of the first-digit proportion throughout the period under analysis. Before specifying our statistical model, we first test the overall validity of Benford’s Law in the GTC dataset. Fig 2 shows the proportion of first digits in the GTC dataset against Benford’s Law. The first-digit proportions in the pooled GTC data resemble the probability mass from Benford’s Law. In Fig 2, digit 1 and digit 6 to digit 9 exhibits higher proportion than the probability from Eq (1) among all digits, whereas digits 2 to 4 present lower than the counterpart values. We discuss the variation of each proportion in detail with our time-varying model.

### Modeling time-varying conformance to Benford’s Law

#### Model specification

We construct a smooth multinomial model which will capture the time-varying proportions in the leading digits and compare the variation with Benford’s distribution. Our GTC dataset is composed of two records for each cyclone: the first digit of traveled distance and the year a cyclone was first observed. Let *N*_*t*_ be the number of cyclones occurring in year *t*, and let ***n***_*t*_ = (*n*_1,*t*_, …, *n*_9,*t*_) with *n*_*d*,*t*_ denoting the frequency of cyclones whose first digit of traveled distance equals to *d*, during year *t*. Below, ***n***_*t*_ is assumed to follow a multinomial distribution with parameter

$$\begin{array}{c} (N_{t},p_{1,t},\ldots ,p_{9,t}), \end{array}$$ (2)

where the *p*_*d*,*t*_’s obey $\sum_{d=1}^{9}p_{d,t}=1$, for all *t*.

Our primary interest is in the probability ***p***_*t*_ = (*p*_1,*t*_, …, *p*_9,*t*_), that is the probability of occurrence of each digit at year *t*; we will refer to ***p***_*t*_ as the *first-digit probability*. More precisely, our target below will be on learning from the data about the dynamics governing the first-digit probability, ***p***_*t*_, and on contrasting it with Benford’s Law, p_*d*_, in Eq (1).

Since our data is composed of frequencies of nine digits together with time *t*, it is natural to relate the first-digit probability to the time predictor via a generalized linear model [13]. We consider a multinomial logistic model where elements of ***p***_*t*_ are connected to a vector of time predictor ***η***_*t*_ = (*η*_1,*t*_, …, *η*_8,*t*_) by

$$\begin{array}{c} p_{d,t}=\frac{\exp(\eta_{d,t})}{1+\sum_{i=1}^{8}\exp\left(\eta_{i,t}\right)},\;d=1,\cdots ,8, \end{array}$$ (3)

and *p*_9,*t*_ is inferred from $\sum_{d=1}^{9}p_{d,t}=1$. Time-varying conformance to Benford’s Law will then be assessed by contrasting *p*_*d*,*t*_ as in (3) against the benchmark p_*d*_, from Eq (1).

To trace the dynamics governing ***p***_*t*_, we employ degree 3 B-spline basis [14], also known as cubic splines, which produce a smooth curve for each element of ***η***_*t*_; cubic splines are the standard choice in the literature as they are twice continuously differentiable and thus allow for a reasonable amount of smoothness [15]. We assume that the B–spline basis functions have *K*+ 1 equally spaced knots, *t*_min_ = *t*_0_ < *t*_1_ < ⋯ < *t*_*K*−1_ < *t*_*K*_ = *t*_max_ over the entire observation period, and thus the smooth curve *η*_*d*,*t*_ can be expressed by the following linear combination of B–splines,

$$\begin{array}{c} \eta_{d,t}=\sum_{k=1}^{K+3}\beta_{d,k}B_{k}\left(t\right),\;d=1,\ldots ,8. \end{array}$$ (4)

Here the *β*_*d*,*k*_’s are regression coefficients of B-splines predictors for digit *d*, and *B*_*k*_(*t*) is a set of B–splines basis functions of degree 3.

To assess overall conformance over nine digits with the First-Digit Rule in each year, we use the smooth sum of squared deviations (SSD) of each digit as a summary statistic. The smooth SSD is computed by a sum of squares of the individual discrepancies between leading digits, i.e.

$$\begin{array}{c} \text{SSD}\left(t\right)=\sum_{d=1}^{9}{\left(p_{d,t}-p_{d}\right)}^{2}, \end{array}$$ (5)

where *p*_*d*,*t*_ and p_*d*_ are respectively the first-digit probability from (3), and the probability from Benford’s Law from (1). The smooth SSD will be exactly zero when the first-digit probability happens to equal to Benford’s first-digit distribution.

To sum up, the goal of the model is on tracking the dynamics governing the first-digit probability over time, conformance to the benchmark will be assessed via the smooth SSD as in (5), and we next concentrate on discussing how the Bayesian paradigm can be used to learn about ***p***_*t*_ from the data.

#### Bayesian inference

We follow a Bayesian version of the penalised spline approach [16, 17] so as to learn about the first-digit probability ***p**_t_*. We assign a first-order random walk prior to the regression coefficients ***β***_*d*_ = (*β*_1,*d*_, …, *β*_*K*+3,*d*_)^T^, which relate an independent and identical Gaussian error *ε*_*d*_ with mean zero and variance $\tau_{d}^{2}$, that is,

$$\begin{array}{c} \beta_{d,k}=\beta_{d,k-1}+\varepsilon_{d},\;\varepsilon_{d}∼N\left(0,\tau_{d}^{2}\right),\;k=2,\ldots ,K+3; \end{array}$$ (6)

a flat (uniform) prior is set for the initial coefficient *β*_*d*,1_. The first order random walk prior can be represented in a matrix form, ***Fβ***_*d*_ = *ε*_*d*_, where *ε*_*d*_ is a (*K* + 2)-vector of *ε*_*d*_’s and ***F*** is a difference matrix of dimension (*K* + 2, *K* + 3). The ***F*** has a diagonal of 1’s (*i* = *j*), −1’s for the next elements to the diagonal (*i* = *j* + 1), and zero otherwise for the (*i*, *j*)th element with *i* ∈ {1, …, *K* + 2} and *j* ∈ {1, …, *K* + 3}.

The variance $\tau_{d}^{2}$ controls amount of smoothness of *η*_*d*,*t*_—and hence that of *p*_*d*,*t*_—with a lower $\tau_{d}^{2}$ indicating that variability of the next regression coefficient is restricted around the value of the previous coefficient. Accordingly, the conditional probability of the regression coefficients ***β***_*d*_ given $\tau_{d}^{2}$ is given by

$$\begin{array}{c} \pi \left(\beta_{d}|\tau_{d}^{2}\right)\propto \exp(-\frac{1}{2\tau_{d}^{2}}\beta_{d}^{T}K\beta_{d}), \end{array}$$ (7)

where ***K*** is a penalty matrix, ***K*** = ***F***^T^ **F** obtained from the random walk prior in Eq (6). The precision parameters $\tau_{d}^{2}$’s are estimated along with the regression coefficients in the model by assigning an additional prior. We place a diffuse inverse gamma prior $\tau_{d}^{2}∼\text{IG}\left(a_{0},b_{0}\right)$ with two constants *a*_0_ and *b*_0_ and then apply a uniform prior for performing a sensitivity analysis. To ease notation, in what follows we let ***β*** and ***τ*** stand for the set {***β***_1_, …, ***β***_8_} and $\left\{\tau_{1}^{2},\ldots ,\tau_{8}^{2}\right\}$ respectively.

The likelihood of observing ***n*** = {***n***_1_, …, ***n***_*T*_} is given by the product of multinomial probabilities, that is,

$$\begin{array}{c} L\left(\beta \right)=f\left(n_{1},\ldots ,n_{T}|p_{1},\ldots ,p_{T}\right)\propto \prod_{t=1}^{T}\prod_{d=1}^{9}{\left\{p_{d,t}\left(\beta \right)\right\}}^{n_{d,t}}, \end{array}$$ (8)

where *p_d,t_*(***β***) and *n*_*d*,*t*_ are respectively the probability and the realized frequency of digit *d* in year *t*; note that *p*_*d*,*t*_ is connected to the regression coefficients ***β*** via the link function in (3) and the linear predictors *η*_*d*,*t*_ in (4). The model is summarized in Box 1.

**Box 1. Summary description of the fitted Bayesian smoothing model.**

**Bayesian multinomial logistic smoothing model**

$$\begin{array}{cc} \text{(Likelihood)} & \;\left(n_{1,t},\ldots ,n_{9,t}\right)∼\text{Mult}\left(N_{t},p_{1,t},\ldots ,p_{9,t}\right),\\ \text{(Model}\;\text{Specification)} & \;p_{d,t}=\frac{\exp(\eta_{d,t})}{1+\sum_{d=1}^{8}\exp\left(\eta_{d,t}\right)},\;p_{9,t}=\frac{1}{1+\sum_{d=1}^{8}\exp\left(\eta_{d,t}\right)},\\ & \;\eta_{d,t}=\sum_{k=1}^{K+3}\beta_{d,k}B_{d,k}\left(t\right),\\ \text{(Random}\;\text{Walk}\;\text{Prior)} & \;\beta_{1,d}∼U\left(c_{0},d_{0}\right),\;\beta_{k+1,d}=\beta_{k,d}+\varepsilon_{d},\;\varepsilon_{d}∼N\left(0,\tau_{d}^{2}\right),\\ \text{(Hyper-Prior)} & \;\tau_{d}^{2}∼\text{IG}\left(a_{0},b_{0}\right). \end{array}$$

Bayesian inference is based on the joint posterior distribution given by

$$\begin{array}{c} p\left(\beta ,\tau^{2}|n\right)\propto L\left(\beta \right)\;\pi \left(\beta |\tau^{2}\right)\;\pi \left(\tau^{2}\right), \end{array}$$ (9)

where $\pi \left(\tau^{2}\right)=\prod_{d=1}^{8}\pi \left(\tau_{d}\right)$, with *π*(*τ*_*d*_) denoting the density of an inverse gamma distribution with parameters (*a*_0_, *b*_0_), and $\pi \left(\beta |\tau^{2}\right)=\prod_{d=1}^{8}\pi \left(\beta_{d}|\tau_{d}^{2}\right)$ with $\pi (\beta_{d}|\tau_{d}^{2})$ as in (7). We calculate a full conditional distribution for the regression coefficients ***β*** and ***τ***^2^ from Eq (9),

$$\begin{array}{c} p\left(\beta |n,\tau^{2}\right)\propto L\left(\beta \right)\;\pi \left(\beta |\tau^{2}\right),\;p\left(\tau^{2}|n,\beta \right)\propto \pi \left(\beta |\tau^{2}\right)\;\pi \left(\tau^{2}\right). \end{array}$$ (10)

Since the full conditional distribution p(***β***∣***n*, *τ***^2^) in Eq (10) does not result in a closed form, a natural option to generate posterior samples is to resort to a Metropolis–Hastings algorithm with iteratively weighted least-squares (IWLS) proposals [18, 19]. In practice, a version of our model can be readily implemented with the aid of existing statistical software. The S1 File includes examples with R code.

## Results

We now apply our smooth multinomial logistic model to the GTC data. The masterplan of this section is as follows: first, we learn about the dynamics of the first-digit probability; second, we examine conformance of the first-digit probability to Benford’s Law, and assess the homogeneity within the dataset over the observation period; third, we further examine evidence on the behavior of the second-digit probability. To streamline the comparisons with [8], below we partition the time horizon into two periods (S1: 1842–1960; S2: 1960–2010).

### Dynamics of the first-digit probability

**Fig 3:**
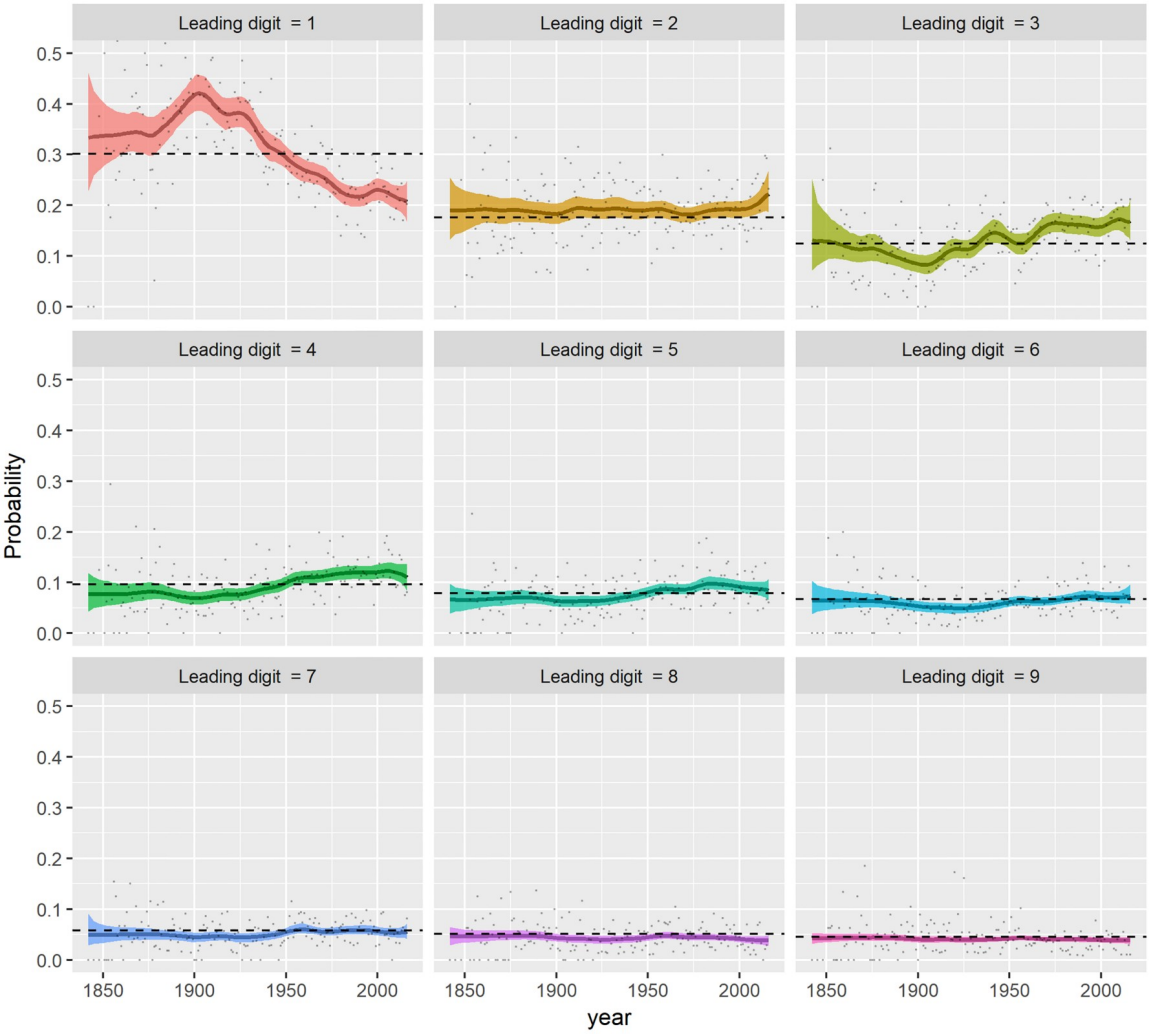
Dynamics of the time-varying first-digit probability *p*_*t*_. Time-varying first-digit probability for digits 1 to 9 (*p*_1,*t*_, …, *p*_9,*t*_) are presented from top left to bottom right. The chart further includes the posterior mean (solid line) and 95% credible bands (shaded areas) of *p*_*d*,*t*_, the sample empirical distribution (point), and Benford’s distribution (dashed line).

We present the dynamics of the probability ***p***_*t*_ in Fig 3. The posterior mean of *p*_*d*,*t*_ and the 95% credible band is compared to the corresponding probability from Benford’s Law, along with the empirical distribution on each panel. The dynamics of posterior distributions of *p*_*d*,*t*_’s show different patterns over the period between leading digits. As expected, we see that in the very early stage of the dataset, e.g. around the 1850s, the corresponding credible bands are much wider than those in the period of 1900s onward due to small sample sizes (see Fig 2). Among all the nine curves, the probability of leading digit one *p*_1,*t*_ has a pronounced variation over the entire period. The posterior mean of *p*_1,*t*_ rises to around 0.4 until the early 1910s, and then steadily drops for more than a century to around 0.2. This implies that the proportion of cyclones whose traveled distance start with digit one decreased approximately by half from around 1900s to recent years. On the contrary, the dynamics of the probability of leading digit three, i.e. *p*_3,*t*_ moves upward the benchmark around the same period as the downward move of leading digit one, although the magnitude of the move is much smaller than that of digit one. The other seven curves move more tightly around the straight line of Benford’s Law, but digit two to digit seven are slightly upward and the others downward the benchmark. Given the variances of the digit probabilities, it is possible that these probabilities stay constant over the observation period S1.

### Time-varying conformance to Benford’s Law

**Fig 4:**
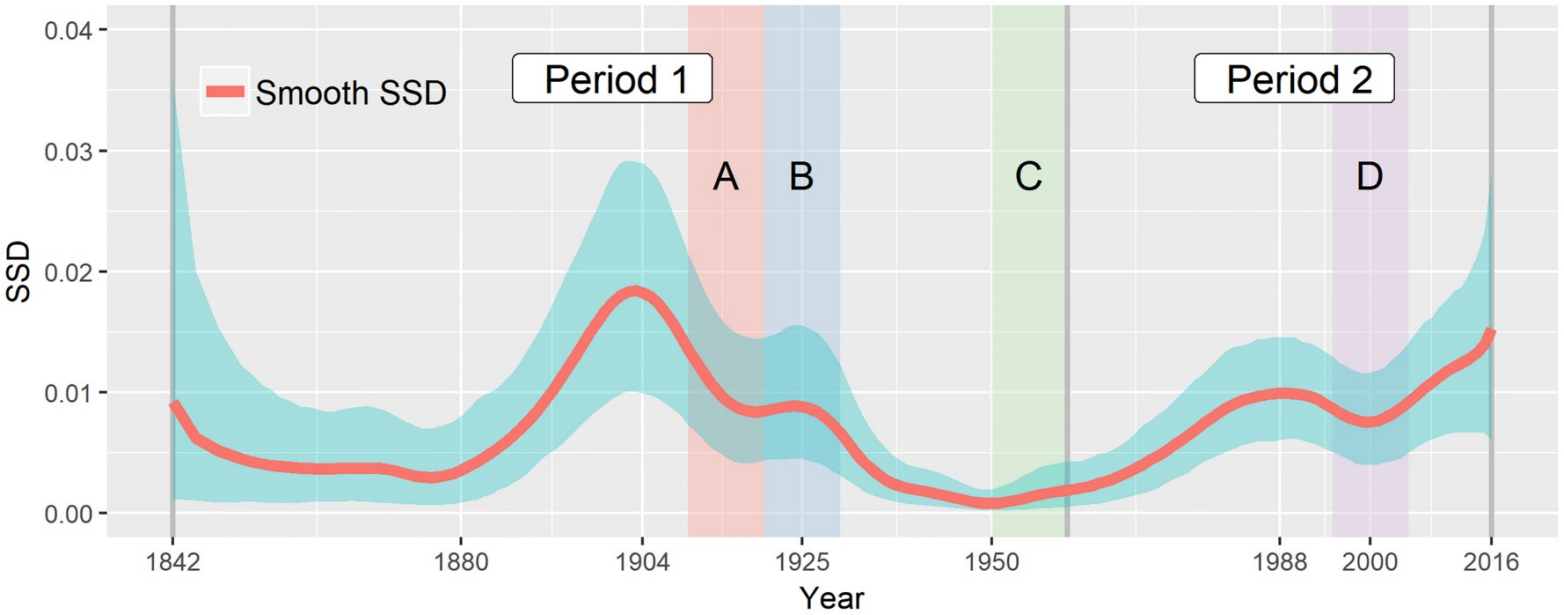
Dynamics of sum of squared deviations (SSD). The chart gives the posterior mean of SSD (solid blue line) and 95% credible bands (shaded blue area) in each year. The time horizons suggested in the previous study is labeled for reference: Two long-term division (Period 1 and 2) and four short-term episodes (Episode A, B, C, and D).

We now turn to time-varying conformance of the first-digit probability to Benford’s Law. To assess overall conformance over nine digits with the First-Digit Rule in each year, we resort to the smooth SSD statistics from Eq 5. Fig 4 depicts the posterior mean and the 95% credible band of the smooth SSD. As with the first digit probability, the SSD also reflects uncertainty from different sample sizes and intrinsic variability of *p*_*d*,*t*_’s.

The smooth SSD avoids overestimation of the misfit due to a discretization effect, whereas a naive empirical SSD as in [8] can be shown to be biased. As the numerical experiments in the S1 File illustrate, the empirical SSD can provide a biased and misleading snapshot of conformance to Benford’s Law (see Fig 4, S1 File). For the GTC dataset, the empirical SSD (not reported) would be generally well above the smooth SSD curve from Fig 4, especially in the years where the number of cyclones was lower.

The smooth SSD examines the heterogeneity within the dataset over time in terms of Benford’s Law. Our results reject the hypothesis of homogeneity across the entire period of observation, as no horizontal line would fit the credible band of the smooth SSD. For the early decades prior to 1880s, the smooth SSD is susceptible to considerable variability due to small sample size, and hence it is difficult to tell either conformance or lack of conformance. However, ever since then, the posterior mean of the smooth SSD starts soon to increase gradually from the 1880s, reaches a peak value of 0.0184 in 1903, and then returns to a lower level around 1940, which constitute the first long-term cycle in the variation of the smooth SSD. Another substantive long-term deviation is currently in progress since the 1970s. The first peak occurs in 1989 with the posterior mean 0.00995 and then the mean falls slightly to 0.00757, ending up with the highest value of 0.0153 in 2016. As shown in Fig 4, the second period has a large SSD value for the first period in magnitude, and hence these periods represent two different heterogeneity in the dataset.

To streamline comparisons, Fig 4 includes the sub-period division of [8]: Episode A and C show periods of decreasing misfit, which was claimed to be explained by technical advancements of collecting and coordinating data as a result of the introduction of telegraph lines and aircraft; Episode B, a sudden rise in a downward trend, was claimed to be possibly due by potential climate variation such as El Nino Southern Oscillation (ENSO); a small bump of misfit during Episode D was claimed to be possibly explained by a mix of effects of new technology and potential climate change.

**Fig 5:**
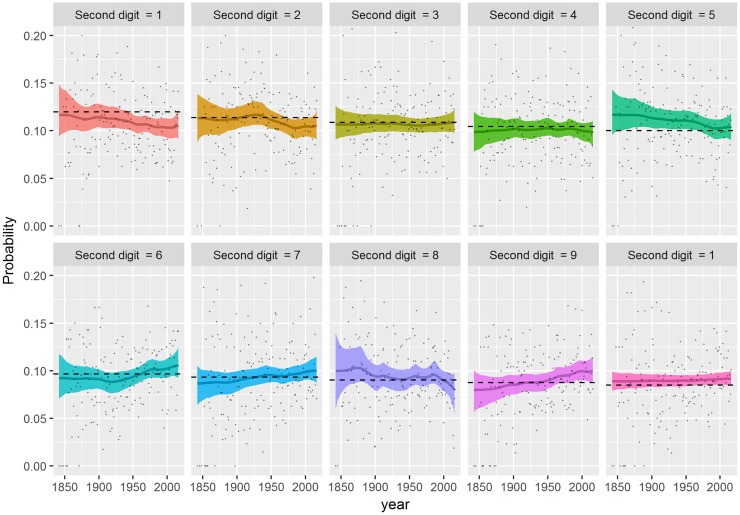
Dynamics of the time-varying second-digit probability $p_{t}^{\left(2\right)}$. Time-varying second-digit probability for digits 0 to 9 $\left(p_{0,t}^{\left(2\right)},\ldots ,p_{9,t}^{\left(2\right)}\right)$ are presented from top left to bottom right. The chart further includes the posterior mean (solid line) and 95% credible bands (shaded areas) of $p_{d,t}^{\left(2\right)}$, the sample empirical distribution (point), and Benford’s distribution (dashed line).

Despite the conclusion of [8] that the GTC data tend to conform to Benford’s Law from 1960 onward, our model actually finds a substantial deviation from Benford’s Law over that period. Keeping in mind that technological improvements are cumulative, we find that the most recent heterogeneity levels actually tend to be higher than the ones from 1842 to 1890 (cf Fig 4). This finding seems to be contradiction with [8] (cf Fig 5 in their paper), which is possibly due to the above-mentioned bias issue faced by their approach.

### Evidence from the second-digit analysis

We further examine the second-digit probability $p_{t}^{\left(2\right)}=\left(p_{0,t}^{\left(2\right)},\ldots ,p_{9,t}^{\left(2\right)}\right)$ in the GTC dataset. Benford’s Second-Digit Rule is given by

$$\begin{array}{c} p_{d}^{\left(2\right)}=\text{P}\left(D_{2}=d\right)=\sum_{k=1}^{9}\log_{10}(1+\frac{1}{10\cdot k+d}),\;d=0,\ldots ,9, \end{array}$$ (11)

where *D*_2_ is the second significant digit of a random variable [20]. Fig 5 illustrates the dynamics of the second-digit probability $p_{t}^{\left(2\right)}$. The dynamics of each $p_{d,t}^{\left(2\right)}$ yields either gradually increasing or decreasing linear trends over the entire period, but the variation of each digit is mostly contained within the credible bands except for digit zero and digit four.

**Fig 6:**
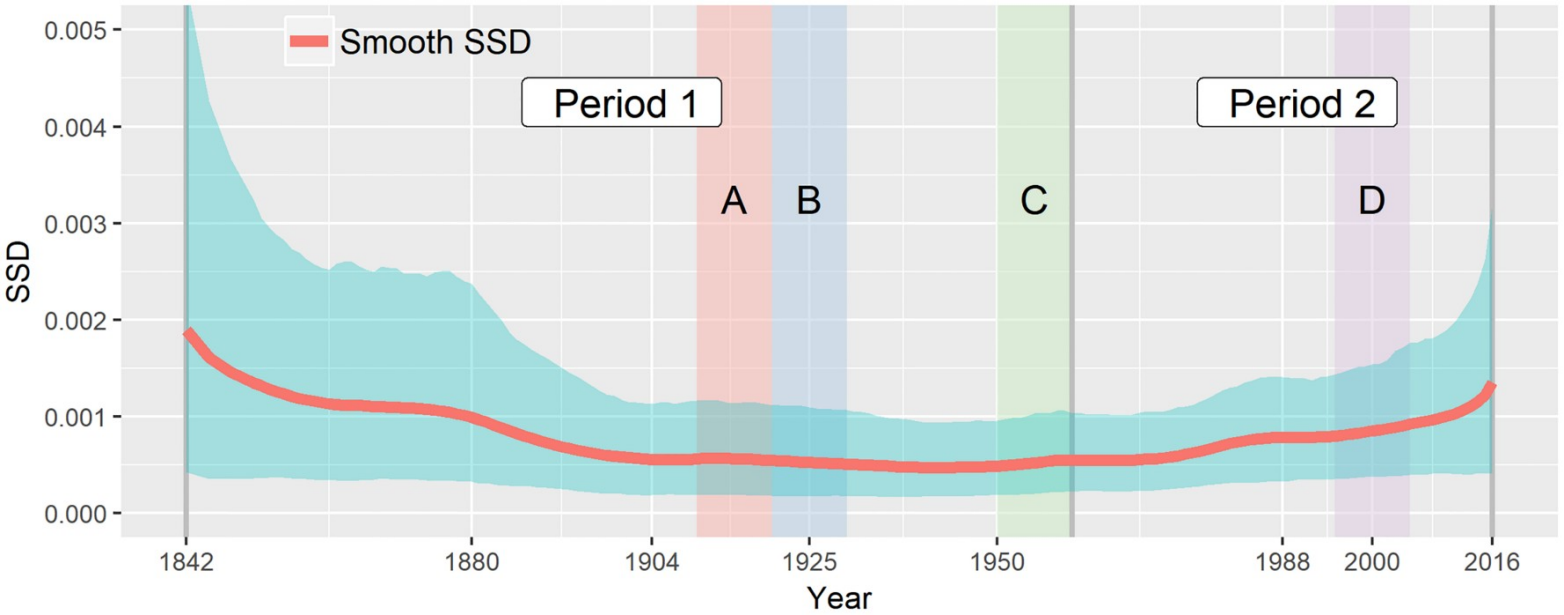
Dynamics of the second-digit SSD. The chart presents the posterior mean of the second-digit SSD (solid blue line) and 95% credible bands (shaded blue area) in each year. The time horizons are labeled for reference as in the first-digit analysis.

We also examine overall time-evolving conformance to Benford’s Law between ten digits. The distribution of the second-digit smooth SSD is obtained from Benford’s Second-Digit Rule in Eq (11) and the posterior sample of the second-digit probability, and presented in Fig 6. The posterior mean of the second-digit SSD starts from high levels and dwindles until around 1950s, then gradually increasing up to recent years. The posterior mean of $p_{t}^{\left(2\right)}$ gives consistent results to our finding in the first-digit analysis that the heterogeneity of the dataset may have been increasing recently.

## Closing remarks

This paper devises a smooth Bayesian model based on penalized splines so to track time-varying conformance to Benford’s law. We have explored the dynamics of the first- and second-digit probability to test the homogeneity of the GTC dataset by comparing the variation with Benford’s Law. Our model enables us to track directly spans of years at which conformance to Benford’s Law is lower, and therefore facilitates the statistical inference about the intrinsic distribution of the first or second digits by evaluating discrepancies from Benford’s Law. Numerical studies in the S1 File show that our method avoids pitfalls faced by pointwise empirical approaches. With respect to our empirical findings versus those of [8]. There seems to be a consensus that the heterogeneity up to early 20th century could be mainly induced by the incomplete management of cyclone records and inevitable measurement errors. Technological developments in the 20th century have enable meteorologists to detect even tiny cyclones and to precisely locate the tracks of those cyclones, which results in the consistently increasing number of cyclones until the 1970s. Our results suggest that heterogeneity starts increasing again, even though the frequency of cyclones has been stable since the 1970s. While technological improvements are cumulative we find that the most recent heterogeneity levels actually tend to be higher than the ones from 1842 to 1890 (see Fig 4); this finding seems to contradict [8] (cf Fig 5 in their paper), possibly due to the above-mentioned bias issue.

While we have centered the paper on the tropical cyclone application, our Bayesian time-varying approach has the potential to be applied in other setups where the goal is on inferring the dynamics governing conformance to Benford’s Law—including fraud analysis.

## Supporting information

S1 File Supplementary materials. (PDF)

## References

[1] Newcomb S. Note on the frequency of use of the different digits in natural numbers. Am J Math. 1881;4(1):39–40. 10.2307/2369148

[2] Benford F. The law of anomalous numbers. Proc Am Phil Soc. 1938;78(4):551–572.

[3] Hill TP. A statistical derivation of the significant-digit law. Statist Sci. 1995;10(4):354–363. 10.1214/ss/1177009869

[4] Miller SJ. Benford’s law: theory & applications. Princeton NJ: Princeton University Press; 2015.

[5] Tsagbey S, de Carvalho M, Page GL. All data are wrong, but some are useful? Advocating the need for data auditing. Am Statist. 2017;71:231–235. 10.1080/00031305.2017.1311282

[6] Ley E. On the peculiar distribution of the U.S. stock indexes’ digits. Am Statist. 1996;50(4):311–313. 10.2307/2684926

[7] Corazza M, Ellero A, Zorzi A. Checking financial markets via Benford’s law: the S&P 500 case In: Corazza M, Pizzi C, editors. Mathematical and Statistical Methods for Actuarial Sciences and Finance. Milano: Springer Milan; 2010 p. 93–102.

[8] Joannes-Boyau R, Bodin T, Scheffers A, Sambridge M, May SM. Using Benford’s law to investigate natural hazard dataset homogeneity. Scient Rep. 2015;5:12046 10.1038/srep12046 PMC4496784 26156060

[9] R Development Core Team. R: A language and environment for statistical computing. Vienna, Austria: R Foundation for Statistical Computing; 2016.

[10] Emanuel K. Increasing destructiveness of tropical cyclones over the past 30 years. Nature. 2005;436:686 10.1038/nature03906 16056221

[11] Landsea CW. Hurricanes and global warming. Nature. 2005;438:E11 10.1038/nature04477 16371953

[12] Hijmans RJ. geosphere: spherical trigonometry; 2017. Available from: https://CRAN.R-project.org/package=geosphere.

[13] Dobson AJ. An introduction to generalized linear models. 3rd ed Chapman & Hall/CRC. Boca Raton: Chapman & Hall/CRC; 2008.

[14] De Boor C. A practical guide to splines; rev. ed Applied mathematical sciences. Berlin: Springer; 2001.

[15] Fahrmeir L, Kneib T. Bayesian smoothing and regression for longitudinal, spatial and event history data. Oxford University Press; 2011.

[16] Lang S, Brezger A. Bayesian P-splines. J Comput Graph Statist. 2004;13(1):183–212. 10.1198/1061860043010

[17] Brezger A, Steiner WJ. Monotonic regression based on Bayesian P-splines: an application to estimating price response functions from store-level scanner data. J Bus Econ Statist. 2008;26:90–104. 10.1198/073500107000000223

[18] Gamerman D. Sampling from the posterior distribution in generalized linear mixed models. Statist Comput. 1997;7(1):57–68. 10.1023/A:1018509429360

[19] Brezger A, Lang S. Generalized structured additive regression based on Bayesian P-splines. Comput Statist Data Anal. 2006;50(4):967–991. 10.1016/j.csda.2004.10.011

[20] Diekmann A. Not the first digit! Using Benford’s law to detect fraudulent scientific data. J Appl Statist. 2007;34(3):321–329. 10.1080/02664760601004940

