# Supplementary material for: Technological improvements or climate change? Bayesian modeling of time-varying conformance to Benford’s Law
Source: PLoS One. 2019 Apr 12;14(4):e0213300. doi: 10.1371/journal.pone.0213300 (PMC6461246; doi:10.1371/journal.pone.0213300)
Supplement: S1 File — (PDF) [file pone.0213300.s001.pdf]

Supplementary Materials

This supplement includes numerical experiments showcasing the performance of the methods and R code to implement the proposed approach along with some supporting reports on empirical results and Bayesian inferences.

Numerical Experiments

Simulation Configurations and Preliminary Experiments

To assess the performance of our method, we simulate data from

$$(n_{1,t}, \dots, n_{9,t}) \sim \text{Mult}(30, p_{1,t}, \dots, p_{9,t}), \quad t = 1, \dots, 80, \tag{1}$$

where $(n_{1,t}, \dots, n_{9,t})$ are the joint counts of leading digits with $\sum_{d=1}^{9} n_{d,t} = 30$ at time t, and where we assume that the time-varying first-digit probabilities are

$$p_{d,t} = \log_{10}\left[\frac{1 + 9 \cdot (d/9)^{\theta_t}}{1 + 9 \cdot \{(d-1)/9\}^{\theta_t}}\right], \quad \theta_t = 1 + 0.5 \cdot \sin\left(\frac{t}{10}\right), \quad d = 1, \dots, 9. \tag{2}$$

Note that $\sum_{d=1}^{9} p_{d,t} = 1$, for every t. Fig 1 illustrates the dynamics over time of the true first-digit probability as in (2).

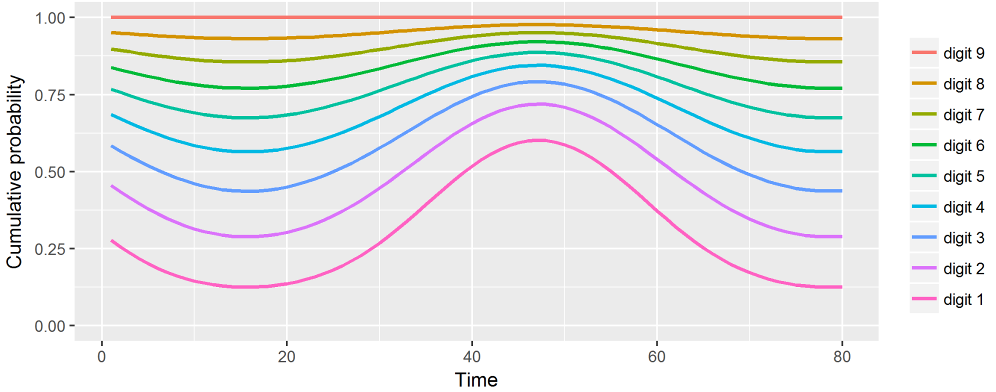

Fig 1. Dynamics of the first-digit cumulative probability. Each line represents the cumulative multinomial probability up to digit d, i.e. $\sum_{i=1}^{d} p_{i,t}$.

First, we concentrate on illustrating the method on a single-run experiment; Monte Carlo evidence is reported in the next section. We generate a random sample from (1),

and then apply our model to obtain posterior distribution of the first-digit probability. We run four chains of size 2,000 using Metropolis–Hastings algorithm with burning-in first 1,000 iteration and thinning 4. Fig 2 depicts the posterior mean of $p_{d,t}$, along with 95% credible bands and the true multinomial probabilities. As can be seen from Fig 2, the posterior mean of $p_{d,t}$ follows closely the true $p_{d,t}$ as defined in (2), and the credible bands tend to include the true $p_{d,t}$. Moreover, if the pooled dataset follows Benford's Law, we can make an inference on when the first-digit probability deviates from the first-digit rule by comparing the posterior distribution of the first digit probability mass with the horizontal line from Benford's Law. For now, the result should be regarded as

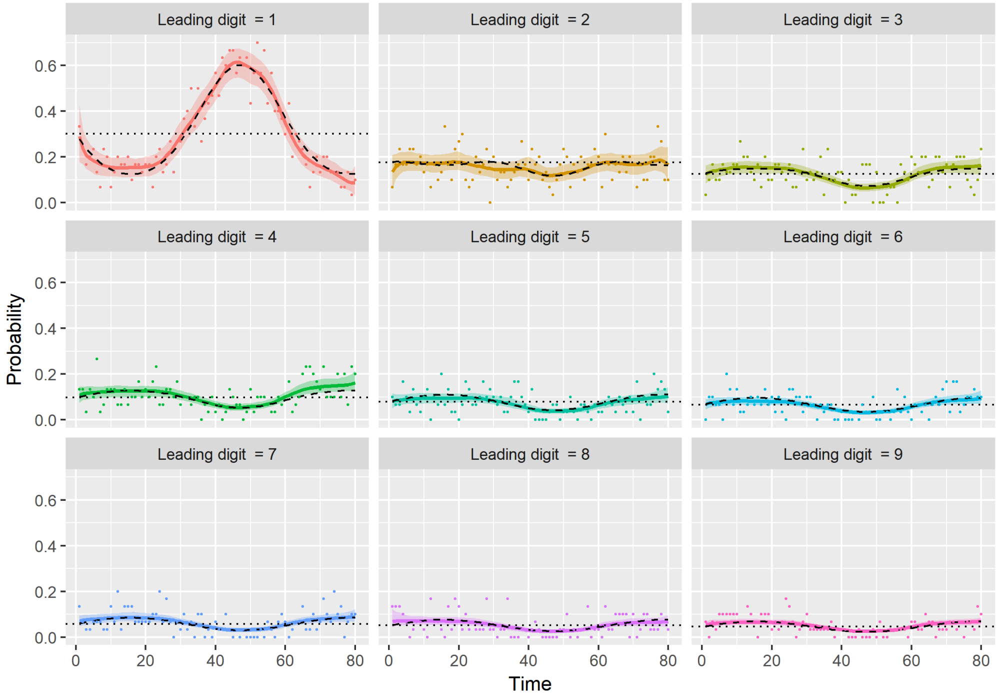

Fig 2. A single-run experiment with data simulated according to (2). On each panel, we represent the posterior mean of $p_{d,t}$ (solid line), the 95% credible bands (shaded area), empirical distribution (points), the true $p_{d,t}$ (dashed line), and the probability mass of Benford's Law (dotted line).

tentative, since Fig 2 summarizes the outcome of a single-run experiment. Next, we assess how robust these findings are over other runs of simulated data.

Monte Carlo Evidence. A Monte Carlo study was conducted by simulating $B = 500$ samples from the model in (1), using the same setting as in the previous section (that is, $N_t = 30$ and $p_{d,t}$ as in (2)). Fig 4 displays trajectories of the posterior means across 500

simulated datasets and their Monte Carlo mean. Our method successfully recovers the corresponding true first-digit probability, in spite of considerable variations of the multinomial probabilities over the period.

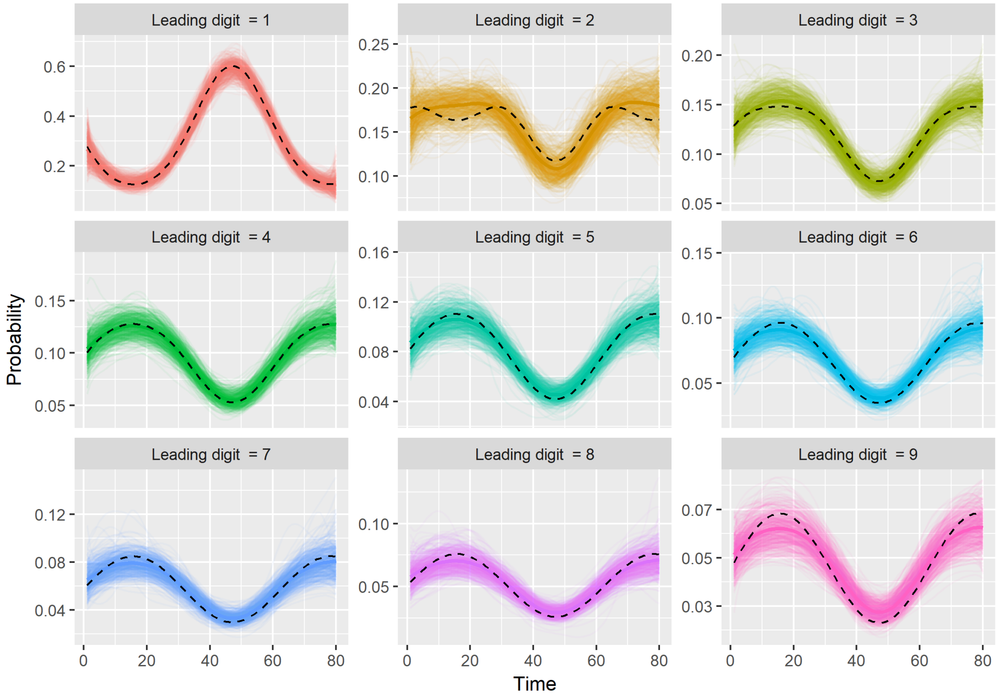

Fig 3. Trajectories resulting from fitting the model on simulated datasets and their Monte Carlo mean. On each panel, we present all the trajectories (translucent lines), the Monte Carlo mean (solid line), and the true $p_{d,t}$ (dashed line).

Discretization Effects. Fig 4 highlights that the empirical-based approach by [1] can suffer from bias.

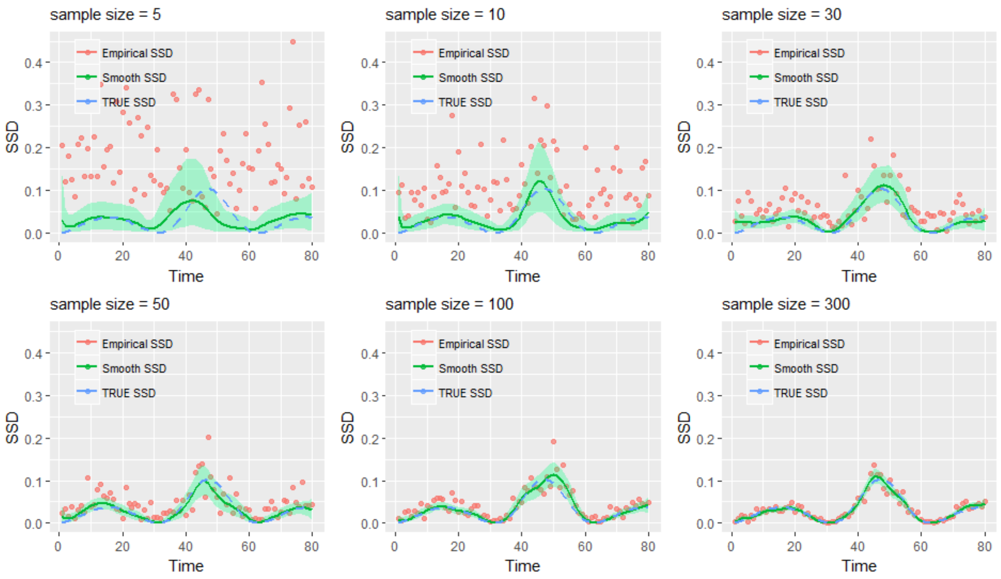

Fig 4. Sum of Squared Deviations (SSD) over six different sample sizes. On each panel, we present the true (blue line), smooth (green line), and empirical (red points) SSD.

R code

In this section, we present R code for implementing the time-varying model used in the Numerical Experiments. The interpretation of the results in the script is discussed in the previous section. In the code chunks below, we follow the 80 characters per line standard. Before running the code chunks, we start by installing the packages `splines2` and `R2jags` (if not installed). The `splines2` package yields B-splines basis functions and the `R2jags` package implements a Metropolis–Hastings algorithm by calling `JAGS` (Just Another Gibbs Sampler), a statistical software for Bayesian data analysis.

```
## Install required packages
packages <- c("R2jags", "splines2")
new <- packages[!(packages %in% installed.packages()[, "Package"])]
if (length(new)) install.packages(new)
## Load required packages
sapply(packages, require, character.only = TRUE)

##   R2jags splines2
##     TRUE     TRUE
```

Next, we define the true time-varying first-digit probability in (2) and then generate multinomial random vectors in (1) at time t using the `rmultinom` function. The seed (`set.seed`) is fixed below for reproducibility reasons.

```
## Define the true time-varying first-digit probability
t <- 1:80 # time span
d <- 1:9 # digits
N <- 30 # number of realizations at each time
theta <- 1 + 0.5 * sin(t / 10)
prob <- matrix(0, nrow = 80, ncol = 9)
for (i in t) {
  prob[i, ] <- log10(1 + 9 * (d / 9)^theta[i]) - log10(1 + 9 * ((d - 1) / 9)^theta[i])
}
## Generate a sample from the true time-varying probability
set.seed(789)
y <- matrix(0, nrow = 80, ncol = 9)
for (j in t) y[j, ] <- rmultinom(1, size = N, prob[j, ])
```

We then set the number of knots and compute B-spline predictors, and set the penalty matrix to use penalized splines.

```r
## Setting up penalized splines
no.in.knots <- 15 # number of internal knots
in.knots <- quantile(t, # Generate equi-distant knots
                        probs = (1:no.in.knots) / (no.in.knots + 1), type = 1)
Bsp <- bSpline(t, knots = in.knots, degree = 3, intercept = TRUE)
Dd <- cbind(diag(length(in.knots) + 3), 0) - cbind(0, diag(length(in.knots) + 3))
Kmat <- t(Dd) %*% Dd # Penalty matrix
```

The following code chunks are used for calling and implementing our method in `JAGS`. In R, we can write the model in BUGS language and specify parameters, initial values, and data. The command `jags` connects inputs in R to `JAGS` and saves the simulations for easy access in R.

```r
## Define objects for JAGS software
# JAGS model (BUGS language)
model <- function() {
  for (l in 1:8) {
    beta[1, l] ~ dnorm(0, 0.0001) # prior for beta1
    for (m in 2:(no.in.knots + 4)){ # random walk priors for beta's
      beta[m, l] <- beta[m - 1, l] + u[m - 1, l]
      u[m - 1, l] ~ dnorm(0, tau[l]) }
    tau[l] ~ dgamma(0.0001, 0.0001) } # prior for tau's
  for (i in 1:80) { # likelihood
    y[i, 1:9] ~ dmulti(pt[i, 1:9], N)
    for (j in 1:8) {
      eta[i, j] <- inprod(Bsp[i, ], beta[, j])
      eeta[i, j] <- exp(eta[i, j])
      pt[i, j] <- exp(eta[i, j]) / (1 + sumeeta[i]) }
    pt[i, 9] <- 1 / (1 + sumeeta[i])
    sumeeta[i] <- sum(eeta[i, ]) } }
# JAGS initial values for tau's
inits <- list( list(tau = rep(0.5, 8)), list(tau = rep(1, 8)),
                list(tau = rep(2, 8)), list(tau = rep(3, 8)))
# JAGS parmeters
parameters <- c("pt", "tau")
# JAGS data
data <- list("y", "N", "Bsp", "no.in.knots")
## Run JAGS in R
results <- jags(data, inits, parameters, model, n.chains = 4,
                                n.iter = 5000, n.thin = 10, n.burnin = 2500)
```

We now plot the resulting outcomes. Below, we present the empirical distribution as points, the posterior mean as a solid line, the credible bands as a polygon and the true multinomial probability as a dashed line.

```
## Extract MCMC samples
pt.array <- results[["BUGSoutput"]][["sims.list"]][["pt"]]
pt.mean <- apply(pt.array, c(2, 3), FUN = mean)
pt.ci <- apply(pt.array, c(2, 3), quantile, probs = c(0.025, 0.975))
```

```
## Plot the time-varying multinomial probabilities
par(mfrow = c(3, 3), mar = c(4, 4, 1, 0) + 0.5)
for (i in 1:9) {
  plot(t, y[, i] / N, type = "p", pch = 20, xlab = "time",
       ylab = "probability", ylim = c(0, 0.8))
  polygon(c(t, rev(t)), c(pt.ci[1, , i], rev(pt.ci[2, , i])),
          col = rgb(190, 190, 190, 127, maxColorValue=255) , border = FALSE)
  lines(t, prob[, i], lwd = 2, lty = 3)
  mtext(side = 3, text = bquote(leading ~ digit ~ .(i)), line = 0, cex = 0.8)
  lines(t, pt.mean[, i], lwd = 2)
}
```

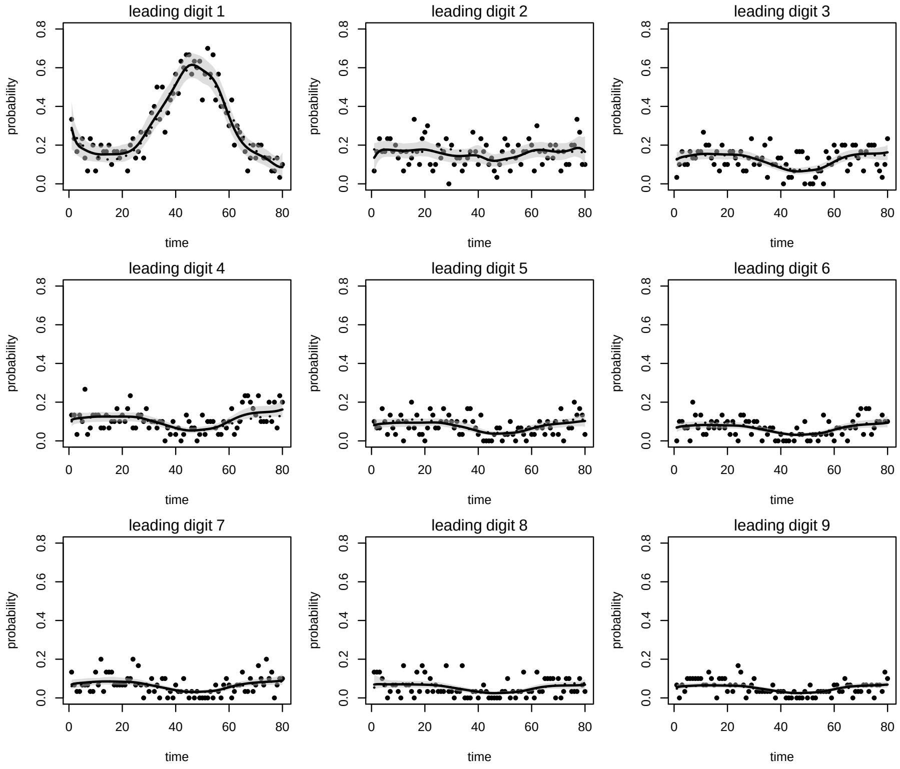

Finally, the code below can be used for comparing the sum of squared deviations (SSD)

among empirical distribution, the first-digit probability, and the true multinomial probabilities.

```
## Calculate SSD's
Ben.prob <- log10(1 + 1 / 1:9)
Ben.matrix <- matrix(Ben.prob, byrow = TRUE, nrow = 80, ncol = 9)
SSD.true <- rowSums((prob - Ben.matrix)^2) # true SSD
SSD.emp <- rowSums((y / N - Ben.matrix)^2) # empirical SSD
dev <- array(NA, dim(pt.array))
for (i in 1:9) {
 dev[, , i] <- pt.array[, , i] - matrix(Ben.prob[i], dim(pt.array)[1], dim(pt.array)[2])
}
SSD.dev <- apply(dev, c(1, 2), FUN = function(x) sum(x^2)) # smooth SSD
SSD.mean <- colMeans(SSD.dev)
SSD.ci <- apply(SSD.dev, 2, quantile, probs = c(0.025, 0.975))
par(mfrow = c(1, 1), mar = c(4, 4, 1, 0) + 0.5)
```

Below, we present the empirical SSD as points, the posterior mean of the smooth SSD as a solid line, the credible bands of the smooth SSD as a polygon and the true SSD as a dashed line.

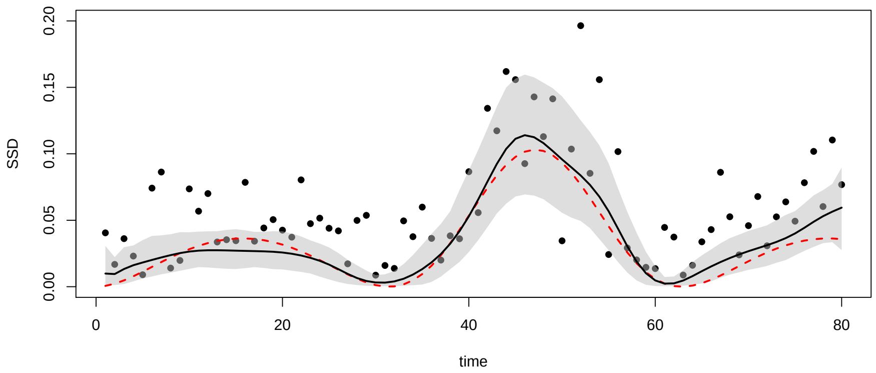

Supporting Reports on Empirical Results and Bayesian Inferences

S Fig. Frequency of Cyclones and Relative Frequency of Traveled Distances

The chart shows the number of tropical cyclones since 1850 and the relative frequency of traveled distances in kilometers in each year.

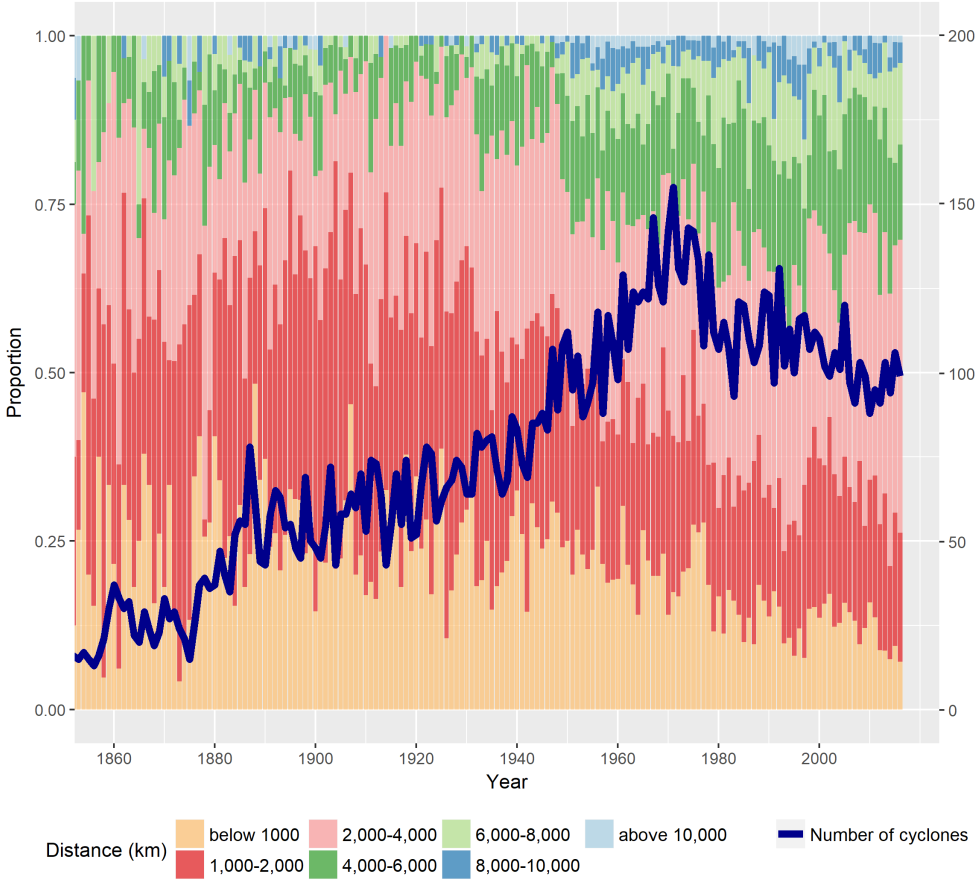

Fig 5. The frequency and relative frequency of traveled distances since 1850. The blue solid line depicts the number of tropical cyclones over time. In each year, the bars show the relative frequency of traveled distances in kilometers.

S1 Fig. Posterior Predictive Checks

This chart shows the posterior predictive distribution for the first-digit probability $\boldsymbol{p}_t$ from our model. As it can be observed, most observed proportions for each digit are covered by the respective 95% credible bands of the predictive distribution, thus suggesting that the model fits well the data.

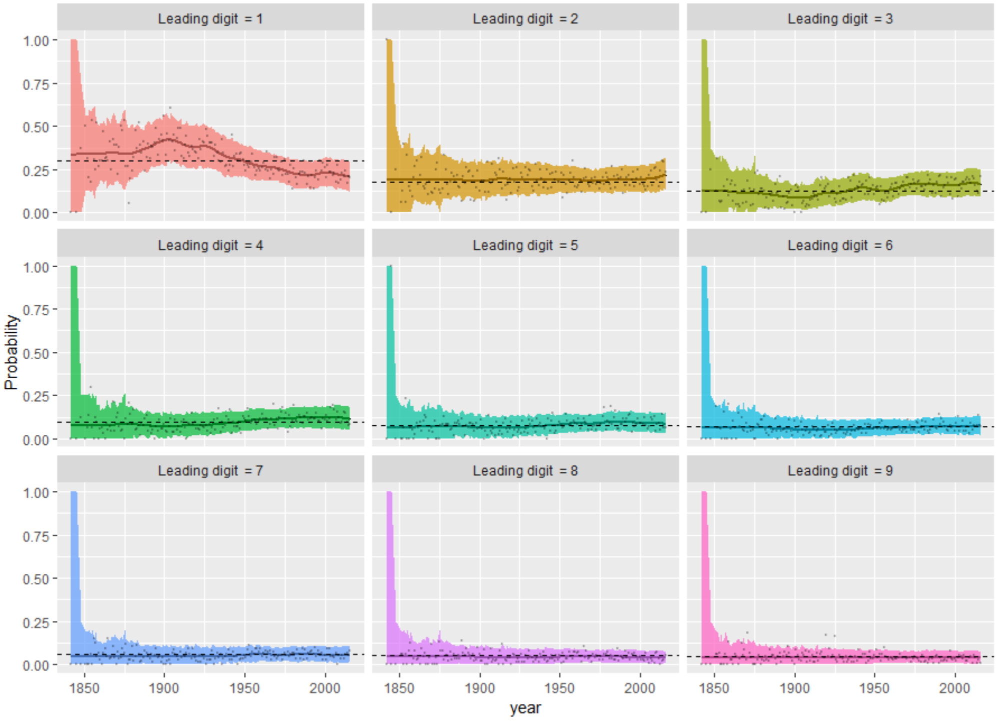

Fig 6. Posterior predictive distribution and model fitting.
The posterior predictive distribution for each digit is presented over the period under analysis. The chart shows the posterior mean (solid line) and 95% predictive credible bands (shaded area), and the sample empirical distribution (point).

S1 Fig. Sensitivity Analysis

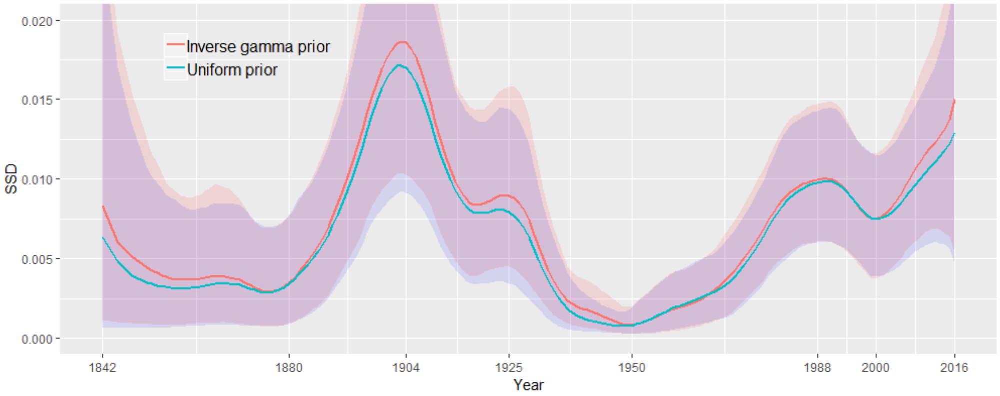

Fig 7. Sensitivity analysis with different priors. The chart compares the dynamics of SSD between two different priors for τ_d. The results from the inverse gamma prior (red) used in the paper are plotted against those from a uniform prior (blue).

References

1. Joannes-Boyau R, Bodin T, Scheffers A, Sambridge M, May SM. Using Benford's law to investigate natural hazard dataset homogeneity. Scient Rep. 2015;5:12046.
